# Integrative Analysis of Long- and Short-Read Transcriptomes Identify the Regulation of Terpenoids Biosynthesis Under Shading Cultivation in *Oenanthe javanica*

**DOI:** 10.3389/fgene.2022.813216

**Published:** 2022-04-07

**Authors:** Kai Feng, Xia-Yue Kan, Rui Li, Ya-Jie Yan, Shu-Ping Zhao, Peng Wu, Liang-Jun Li

**Affiliations:** ^1^ College of Horticulture and Plant Protection, Yangzhou University, Yangzhou, China; ^2^ Joint International Research Laboratory of Agriculture and Agri-Product Safety of Ministry of Education of China, Yangzhou University, Yangzhou, China

**Keywords:** Oenanthe javanica, transcriptome, terpenoids, DEGs, regulation

## Abstract

Water dropwort (*Oenanthe javanica*) is a popular vegetable with high nutritional value and distinctive flavor. The flavor is mainly correlate with the biosynthesis of terpenoids. Shading cultivation was used to improve the flavor in the production of water dropwort. However, the changes of terpenoids and the genes involved in terpenoids biosynthesis under shading treatment remains unclear. In this study, the long- and short-reads transcriptomes of water dropwort were constructed. In total, 57,743 non-redundant high-quality transcripts were obtained from the transcriptome. 28,514 SSRs were identified from non-redundant transcripts and the mono-nucleotide repeats were the most abundant SSRs. The lncRNAs of water dropwort were recognized and their target genes were predicted. The volatile compound contents in petioles and leaf blades of water dropwort were decreased after the shading treatment. The DEGs analysis was performed to identify the terpenoids biosynthesis genes. The results indicated that 5,288 DEGs were differentially expressed in petiole, of which 22 DEGs were enriched in the terpenoids backbone biosynthesis pathway. A total of 12 DEGs in terpenoids biosynthesis pathway were selected and further verified by qRT-PCR assay, demonstrating that the terpenoids biosynthesis genes were down-regulated under shading treatment. Here, the full-length transcriptome was constructed and the regulatory genes related to terpenoids biosynthesis in water dropwort were also investigated. These results will provide useful information for future researches on functional genomics and terpenoids biosynthesis mechanism in water dropwort.

## Introduction

Water dropwort (*Oenanthe javanica*) is a perennial aquatic herb belongs to Apiaceae family, which is mainly cultivated in tropical and temperate regions (Lu and Li, 2019). It is a popular vegetable rich in vitamins, proteins, dietary fibers, and other nutrients (Park et al., 2015; Feng et al., 2018b). Water dropwort contains many bioactive substances and it is well known to have various medicinal effects, such as antithrombotic (Ku et al., 2013), hepatoprotective (Yang et al., 2014), neuroprotective (Ma et al., 2010), anti-inflammatory (Ahn and Lee, 2017), antioxidant (Her et al., 2019), antiviral (Han et al., 2008), and anti-senescence (Moon et al., 2009).

Water dropwort is a vegetable crop with distinctive flavor. It is known that the accumulation of high concentrations of terpenoids in water dropwort produce undesirable flavor (Seo and Baek, 2005). The flavor of water dropwort can be improved by shading cultivation (Ye et al., 2009). In the production of water dropwort, one of the shading cultivation patterns is using the soil to form a soil wall to protect the water dropwort from sunlight. The distinctive flavor and tastes of water dropwort were attributed to the biosynthesis of volatile components. A previous study has reported that the volatile components that affect the taste of water dropwort are mainly terpenoids (Deng et al., 2003). Solid phase microextraction-gas chromatography-olfactometry (SPME-GC-O) assay indicated that the terpenoids, including α-terpinolene, *p*-cymene, β-caryophyllene, and α-terpinene, were the characteristic aroma components in water dropwort (Seo and Baek, 2005). The biosynthesis of terpenoids in water dropwort was controlled by multiple genetic factors, whereas the related regulatory genes remain unknown.

Terpenoids are an important secondary metabolite and one of the most diverse natural products in chemistry and structure (Christianson, 2017). Terpenoids are widely distributed in plants and participate in a variety of physiological processes, such as photosynthesis, ion transport, growth regulation, and stress response (Velikova et al., 2015). Terpenoids also showed high economic value. The β-caryophyllene, linalool, myrcene, and other terpenoids can be used in spices production (Behr and Johnen, 2009; Tholl, 2015). In addition, terpenoids were investigated to have many medicinal functions for humans, including anticancer, anti-inflammatory, and hypoglycemic effects (Amato et al., 2002; Ukiya et al., 2002; Juergens, 2014; Singh and Sharma, 2015). All the terpenoids are synthesized from the common C_5_ isoprene precursors, isopentenyl diphosphate (IPP) or its allylic isomer dimethylallyl diphosphate (DMAPP). The biosynthesis of IPP and DMAPP in plants mainly depends on two pathways, the mevalonate (MVA) pathway located in the cytoplasm and the 2-*C*-methylerythritol 4-phosphate (MEP) pathway located in the plastid (Vranova et al., 2013). The *trans*/*cis*-prenyltransferases catalyzes the conversion of IPP and DMAPP to polyprenyl diphosphates, such as geranyl/neryl diphosphate (GPP/NPP, C_10_), farnesyl diphosphate (FPP, C_15_), and geranylgeranyl diphosphate (GGPP, C_20_). Then, the diverse terpene backbones, including isoprene, monoterpenes, sesquiterpenes, diterpenes, and sesterterpenes, were synthesized under the catalysis of terpene synthase (TPS) (Nagegowda and Gupta, 2020). These terpene backbones are modified by a series of glycosylation, methylation, and hydroxylation to form different terpenoids (Pateraki et al., 2015).

The distinctive aroma and taste of water dropwort was related to the terpenoids biosynthesis and accumulation (Seo and Baek, 2005). The information on molecular mechanism and biosynthesis pathway of terpenoid in water dropwort was still limited. The transcriptome sequencing is considered to be an efficient technology to investigate the gene regulatory network and molecular mechanism in plants (Yang et al., 2012). The next generation sequencing (NGS) has been conducted to identify the genes and miRNA in water dropwort under abiotic stress (Jiang et al., 2015). With the development of sequencing technology, the genome of *O. javanica* was also released recently (Liu et al., 2021). In view of the full-length sequences and transcript structures, the PacBio single molecule real-time (SMRT) technology gradually takes the place of NGS sequencing and become the mainstream of transcriptome sequencing (Li et al., 2017). In this study, the PacBio SMRT and Illumina RNA sequencing was employed to investigate the regulatory genes of terpenoid biosynthesis in water dropwort. The results in this study will provide a novel insight into the terpenoid biosynthesis in water dropwort and give a reference for improving the flavor by molecular breeding.

## Materials and Methods

### Plant Materials and Treatments

“Liyang baiqin,” a water dropwort variety in China, was used as plant material in this study. The plants of water dropwort were grown in the field of Liyang, Jiangsu province (31°42′N, 119°48′E) under natural growth. The shading cultivation was performed. We used soil to form a barrier for preventing sunlight from water dropwort. After shading cultivation for 30 days, the green petioles (GP), green leaf blades (GL), white petioles (WP), and white leaf blade (WL), were collected for further experiments. Three biological replicates of water dropwort plants were prepared.

### Extraction and Measurement of Volatile Compounds

The volatile compounds of water dropwort were extracted and measured using auto-head space-solid phase microextraction-gas chromatography-mass spectrometry (HS-SPME-GC-MS) method (Seo and Baek, 2005), with some modifications. The collected water dropwort was washed and cut into pieces. A total of 5 g of water dropwort samples were placed in flask applied and 3-heptanol (Sigma-Aldrich) was added. The volatile compound was extracted and collected by placing the SPME fiber (65 μm PDMS/DVB) in the headspace at 50°C for 30 min. The collected volatile compound was then injected to GC-MS (DSQ-II Thermo) equipped with DB-5 mass spectrometry column (30 m × 0.25 mm × 0.25 µm) for further analysis. Helium was used as carrier gas and its constant flow rate was 0.8 ml/min. The oven temperature was set at 50°C for 2 min, raised to 75°C at 5°C/min and keep 3 min, then raised to 155°C at 10°C/min and keep 4 min, and finally raised to 210°C at 5 °C/min and keep 2 min. The mass spectra were performed with 70 eV of ionization energy and 33–350 amu of scan range. The kinds of volatile compounds in water dropwort were identified by comparing the mass spectra with the NIST 05 library. 3-heptanol was used as internal standard, and the relative contents of volatile compounds in water dropwort were calculated based on the peak areas (Seo and Baek, 2005).

### Library Preparation and Transcriptome Sequencing

The total RNA was extracted from the three biological replicates of WP, GP, WL, and GL samples of water dropwort, respectively. The purity and quality of RNA were estimated by Nanodrop, Agilent 2,100, and electrophoresis. A total of 12 Illumina RNA sequencing libraries was constructed using the NEBNext^®^ Ultra™ RNA Library Prep Kit (NEB, Massachusetts, United States) according to the manufacturer’s protocols. The mixed RNA samples, F01 (WP and WL) and F02 (GP and GL), were used to construct the PacBio Iso-Seq library. The full-length cDNA was synthesized by SMARTer™ PCR cDNA Synthesis Kit (TaKaRa, Dalian, China) following the operating instructions. Bluepippin (Sage Science Beverly, MA, USA) was used to screen full-length cDNA fragments and construct cDNA libraries with different sizes (1–2, 2–3, and >3 kb). The constructed libraries were evaluated by Qubit 2.0 Fluorometer and Agilent 2,100. The SMRT sequencing and NGS was performed with Pacific Bioscience RS II (Pacific Biosciences, California, United States) platform and Illumina HiSeq4000 platform (San Diego, CA, United States) at Biomarker Technology Co. (Biomarker, Beijing, China), respectively.

### Data Filtering and *de Novo* Assembly

The raw polymerase reads were filtered and the reads of insert (ROI) were extracted with set parameters (full passes of ≥0 and quality of >0.75). The ROI sequences were classified into FLNC and non-full-length reads (NFL) based on the filtering of cDNA primers and polyA tail signal. The FLNC sequences from the same isoform were clustered into one consensus sequence using SMRT Analysis (v2.3.0) software with iterative clustering for error correction (ICE) algorithm. The Quiver program was used to polish the consensus sequences and generate the high-quality isoforms with an accuracy rate of >99%. The quality and accuracy of Illumina RNA-seq data were detected and the raw reads with adapter and low-quality reads were filtered. Subsequently, the low-quality isoforms were further corrected by the Illumina RNA-seq data using *proovread* (Hackl et al., 2014). The CD-HIT program (identity >0.99) was used to de-redundancy the high-quality isoforms and corrected low-quality isoforms (Li and Godzik, 2006). Finally, the non-redundant transcripts with high-quality were constructed.

### Analysis of AS Events, SSR, and lncRNA

The non-redundant transcripts with high-quality were obtained for further alternative splicing (AS) events, simple sequence repeat (SSR), and long non-coding RNAs (lncRNAs) analysis. The IsoSeq AS *de novo* script was used to identity the AS events (Liu et al., 2017). The FLNC transcripts of water dropwort were clustered by Cogent software (v1.0). The General feature format (GFF) file was constructed by GMAP. The AS events were detected in SUPPA with the default settings (Wu and Watanabe, 2005). MIcroSAtellite identification tool (MISA) was used to conduct the SSR analysis (http://pgrc.ipk-gatersleben.de/misa/). Seven types of SSRs were identified from the sequences of transcriptome, including mono-nucleotide repeats, di-nucleotide repeats, tri-nucleotide repeats, tetra-nucleotide repeats, penta-nucleotide repeats, hexa-nucleotide repeats, and compound SSRs. CREMA and RNAplonc were used to predict the lncRNA in plants (Simopoulos et al., 2018; Negri et al., 2019). In this study, four common computational approaches, including coding potential calculator (CPC) (Kong et al., 2007), coding-non-coding index (CNCI) (Sun et al., 2013), coding potential assessment tool (CPAT) (Wang et al., 2013), and Pfam (Finn et al., 2014), were combined to distinguish the lncRNAs in water dropwort. Based on these four approaches, lncRNAs have been successfully identified from long-reads transcriptomes in many species (Cui et al., 2020; Hou et al., 2021). LncRNAs works through mRNA binding, and the target genes of lncRNAs were predicted by LncTar (Li et al., 2015). As an efficient tool for predicting the target genes of lncRNAs, LncTar has been widely used to identify the targets in many plants, such as rice (Leng et al., 2020), *Gnetum luofuense* (Hou et al., 2021), and *Carex breviculmis* (Teng et al., 2019).

### Gene Function Annotation

The coding sequences (CDS) was identified by TransDecoder (v3.0.0) online software (https://github.com/TransDecoder/TransDecoder/releases) based on the length of open reading frame (ORF), log-likelihood score, and alignments of sequences with Pfam database. The function annotation of non-redundant transcripts was conducted using BLAST software (v2.2.26) against seven databases, including NCBI non-redundant protein database (NR), Swiss-prot, gene ontology (GO), clusters of orthologous groups (COG), eukaryotic orthology groups (KOG), protein family (Pfam), kyoto encyclopedia of genes and genomes (KEGG).

### Phylogenetic Analysis

Using PF03936 and PF01397 as queries, the TPS family genes in water dropwort were identified by HMMER 3.0 software with an expected threshold value <10^−4^ (Eddy, 2011). The TPS family sequences of *Arabidopsis thaliana* was obtained from the previous study (Aubourg et al., 2002). The phylogenetic tree was constructed by MEGA 7.0 using neighbor-joining method with 1,000 bootstraps (Kumar et al., 2016; Chen et al., 2017). The TPS genes of water dropwort were divided into different subfamilies according to the phylogenetic relationships with the known *A. thaliana* TPS proteins.

### DEGs Analysis

The high-quality Illumina sequencing reads were aligned with non-redundant transcripts by Bowtie2 (Langmead and Salzberg, 2012). The expression abundance was quantified by RSEM based on the transcripts per million (TPM) (Li and Dewey, 2011). The identification of differentially expressed genes (DEGs) in different samples were conducted by DESeq R package based on the read counts (Anders and Huber, 2010). The transcripts that meet the set parameters (Fold Change ≥2 and FDR <0.01) are identified as DEGs. The GO enrichment analysis of DEGs was conducted using GOseq-R package (Tian et al., 2016). Kolmogorov-Smirnov test was employed to correct the *p*-value, and the corrected *p*-value (≤0.05) was considered as significantly enriched. The enrichment of DEGs in KEGG pathway were analyzed by KOBAS (Xie et al., 2011). The heatmap were performed based on the TPM values to investigate the expression abundance of DEGs in different samples.

### qRT-PCR Assay

To validate the availability of transcriptome, qRT-PCR assay was conducted to further investigate the expression level of DEGs. The qRT-PCR was carried out based on our previous study (Feng et al., 2018a). The primers of selected DEGs were designed by Primer 6.0 software (Supplementary Table S1). *OjPP2A* was selected as internal reference gene. The relative expression levels of DEGs were calculated according to the 2^−ΔΔCT^ method (Schmittgen and Livak, 2008; Jiang et al., 2014). The expression of *F02.PB35028* in WP sample was used as the calibrator for qRT-PCR analysis.

### Statistical Analysis

Statistical analysis was performed by SPSS 17.0 software. Correlation analysis between gene expression and volatile contents in water dropwort were conducted using the Pearson’s correlation analysis.

## Results

### Volatile Compound Contents of Water Dropwort

**FIGURE 1:**
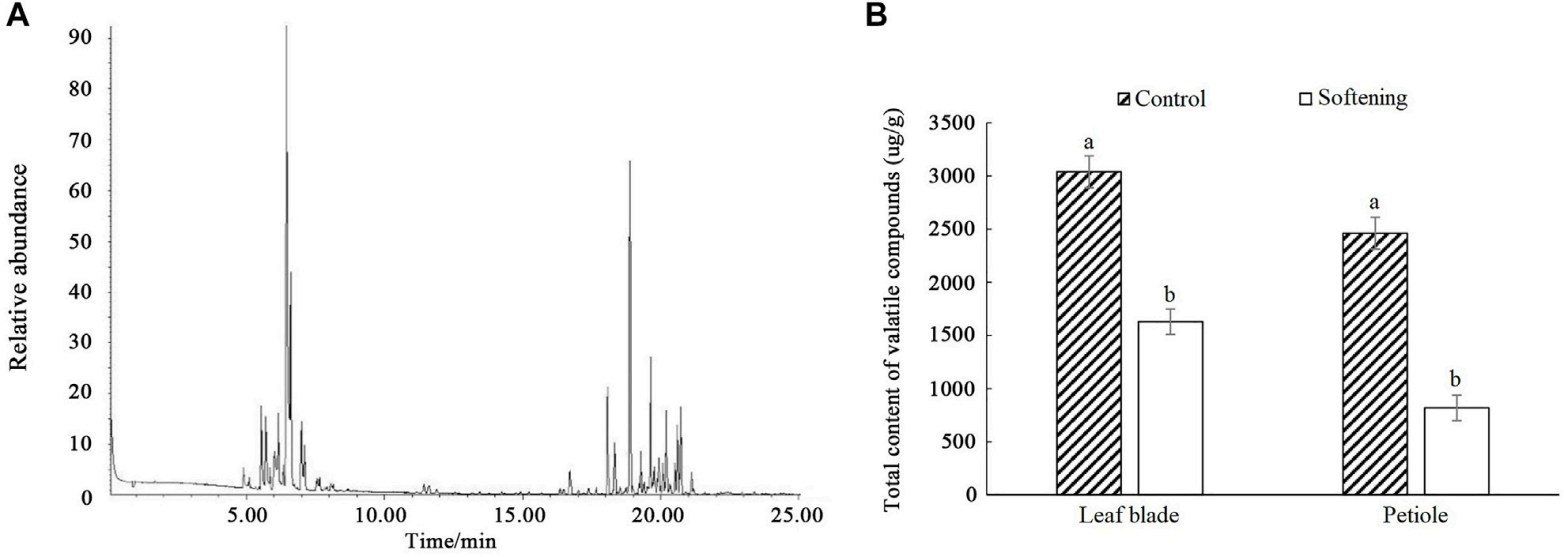
The measurement of volatile compound contents in water dropwort. **(A)** The gas chromatography of volatile compound in water dropwort. **(B)** The total volatile compound contents of water dropwort. The bar represents the mean values of three independent experiments ±SD. Different lowercase letters indicate significant differences at *p* < 0.05.

The volatile compound contents of water dropwort were detected by SPME-GC-MS equipment (Figure 1A). The results indicated that the volatile compounds in water dropwort were mainly composed of terpenoids. The total volatile compound contents of water dropwort were significantly decreased after the shading treatment (Figure 1B).

### Transcriptome Sequencing and Assembly

**FIGURE 2:**
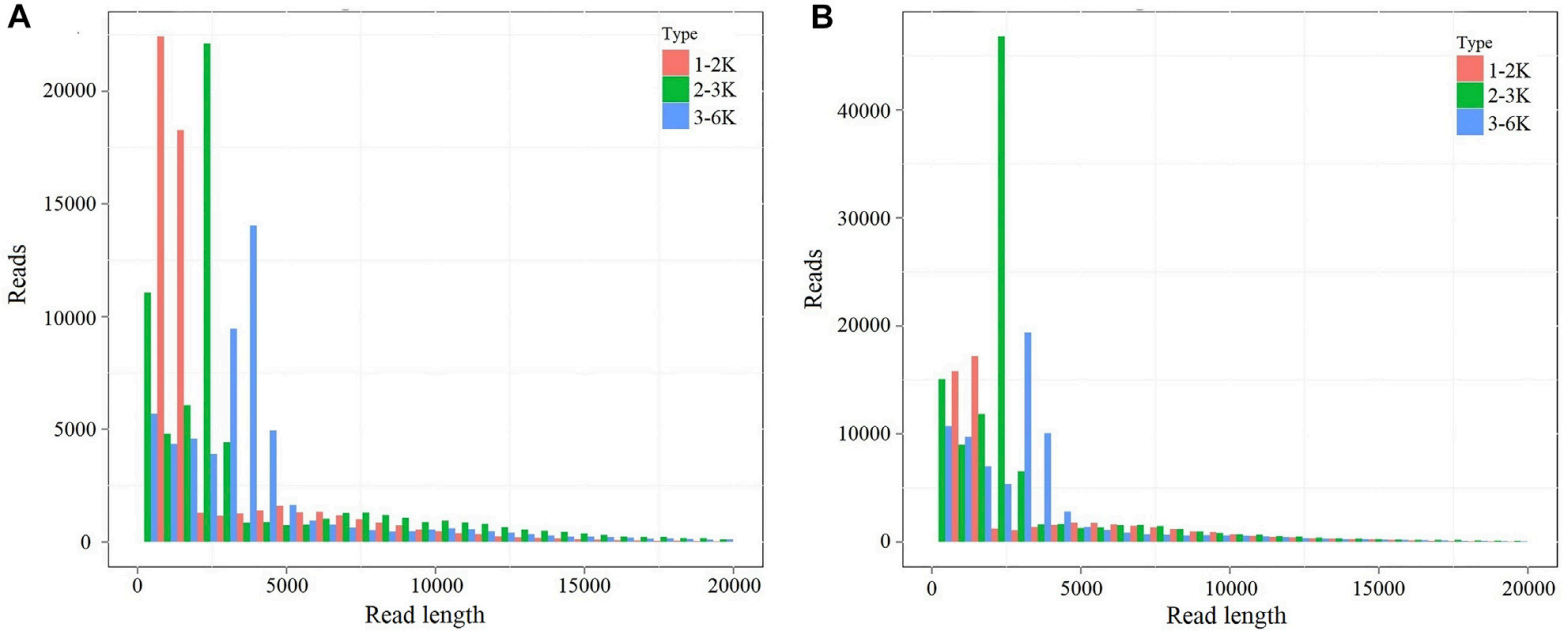
The ROI read length distribution of each size bins. **(A)** The ROI read length distribution of each size bins in F01 sample. **(B)** The ROI read length distribution of each size bins in F02 sample. The columns with different colors indicate different read length.

In this study, two mixed RNA samples (F01 and F02) were used to obtain the full-length transcripts with different libraries sizes (1–2, 2–3, and 3–6 kb). The paired-end reads of different libraries were listed in Supplementary Table S2. A total of 450,876 polymerase read sequences were obtained from F01, and 601,168 polymerase read sequences were obtained from F02. The polymerase reads (length >50 bp and accuracy >0.75) were filtered, yielding 3,539,289 subreads for F01 and 4,203,996 subreads for F02 samples, respectively. The reads of insert (ROI), ROI bases, mean read length of insert, and mean read quality of insert were analyzed in F01 and F02 samples (Supplementary Table S3). The ROI read length distributions of each size bins were shown in Figure 2

For F01 sample, a total of 77,470 FLNCs were extracted from the ROIs. The extracted 47,559 consensus FLNC reads includes 38,186 high-quality isoforms and 9,373 low-quality isoforms. Meanwhile, 103,548 FLNC reads were obtained from F02 sample. The extracted 59,819 consensus FLNC reads were composed of 48,096 high-quality isoforms and 11,723 low-quality isoforms (Supplementary Table S4). Finally, the high-quality and corrected low-quality transcripts of F01 and F02 generated 57,743 non-redundant transcripts.

### AS Events, SSR, and lncRNA Analysis

**FIGURE 3:**
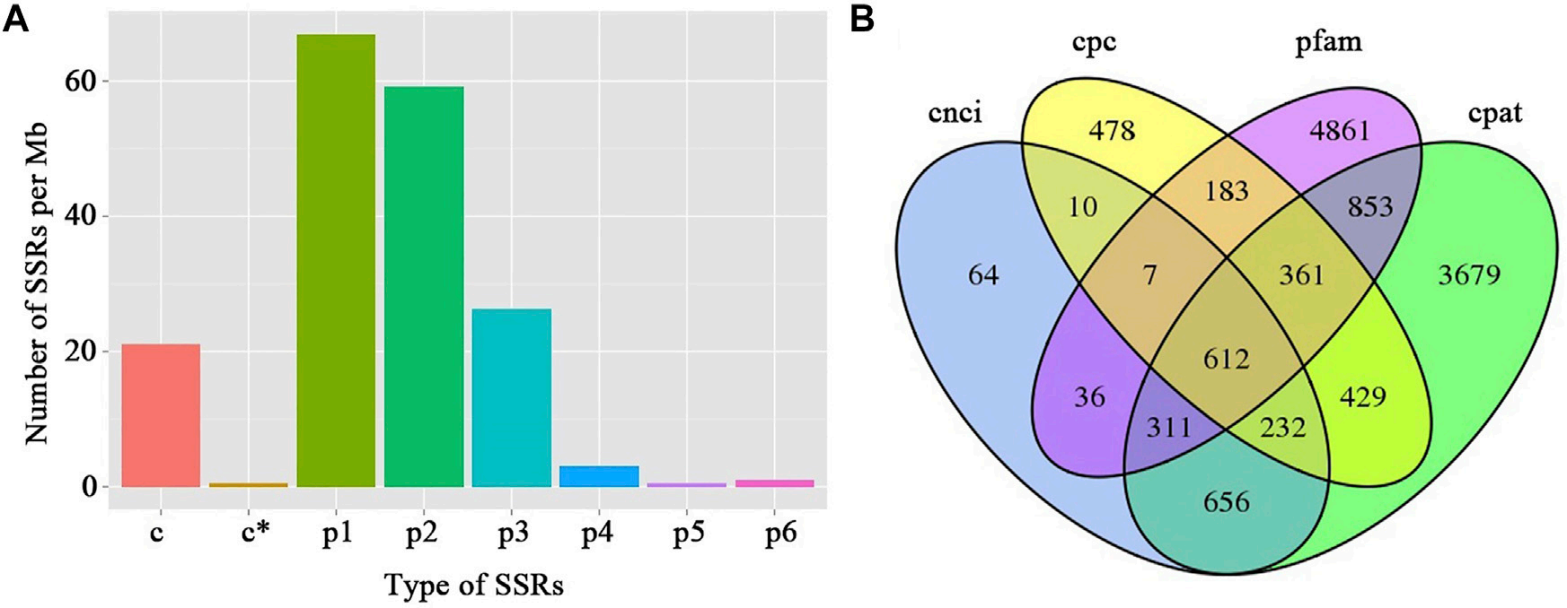
Statistic of SSR density and the prediction of lncRNAs in water dropwort. **(A)** Density analysis of the different SSR types. p1: mono-nucleotide repeats; p2: di-nucleotide repeats; p3: tri-nucleotide repeats; p4: tetra-nucleotide repeats; p5: penta-nucleotide repeats; p6: hexa-nucleotide repeats; c: compound SSR. **(B)** The venn diagram showing the number of lncRNAs using different prediction methods. CPC: coding potential calculator; CNCI: coding-non-coding index; CPAT: coding potential assessment tool; Pfam: protein family.

In total, 664 and 870 AS events were detected from the full-length isoforms of F01 and F02 samples, respectively (Supplementary Table S5). 28,514 SSRs were identified from 57,700 non-redundant transcripts (>500 bp) (Supplementary Table S6). The identified SSRs can be divided into mono-nucleotide repeats (p1), di-nucleotide repeats (p2), tri-nucleotide repeats (p3), tetra-nucleotide repeats (p4), penta-nucleotide repeats (p5), hexa-nucleotide repeats (p6), and compound SSR(c). The density distribution of different SSR types indicated that the most abundant SSRs was mono-nucleotide repeats, followed by di-nucleotide repeats and tri-nucleotide repeats (Figure 3A). CPC, CNCI, CPAT, and Pfam databases were used to predict lncRNA. We found 612 lncRNAs were both present in all four prediction methods (Figure 3B). The target genes predication of lncRNAs demonstrated that 96 lncRNAs were predicted to target at least one gene, among which F02.PB15548 had the maximum target genes with 19 target genes (Supplementary Table S7).

### Coding Sequence Prediction and Functional Annotation

TransDecoder (v3.0.0) was used to predict the coding sequence (CDS) from the non-redundant transcripts. A total of 56,307 open reading frame (ORF) was obtained in water dropwort. The predicted length distribution of the ORF coding protein sequence indicated that the ORFs encoding 200–300 amino acid (aa) was the most, followed by that encoding 100–200 aa and 300–400 aa (Supplementary Figure S1). The transcripts were blast with the NR, Swiss-prot, GO, COG, KOG, Pfam, and KEGG databases to obtain the functional annotation. A total of 57,118 non-redundant transcripts were annotated, among which 27,100 were annotated in GO, 25,644 in KEGG, 37,227 in KOG, 48,433 in Pfam, 43,401 in Swiss-prot, 25,353 in COG, 55,815 in eggNOG, and 56,684 in NR (Supplementary Table S8).

### Phylogenetic Analysis of TPS Family

**FIGURE 4:**
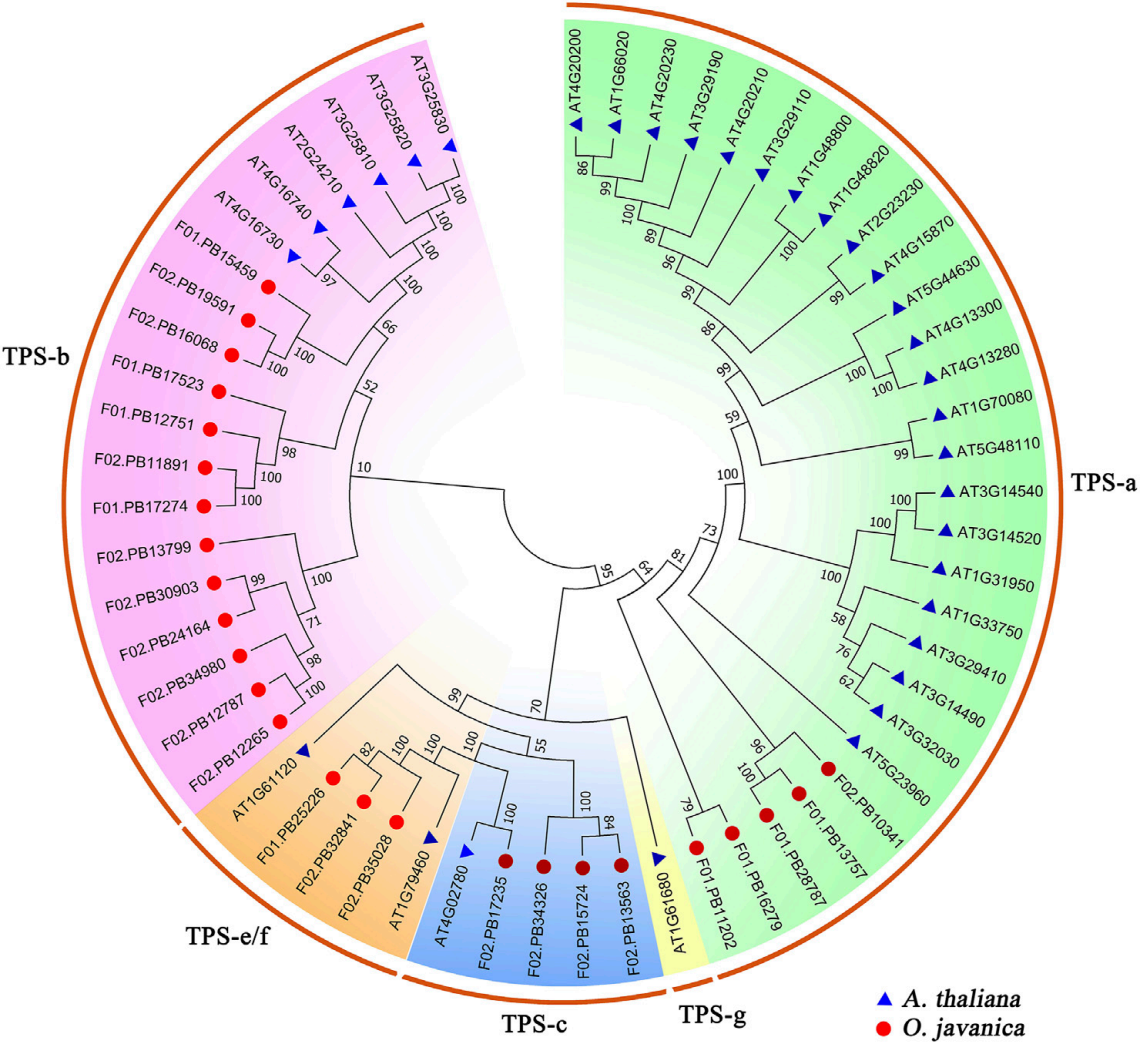
The phylogenetic analysis of TPS family proteins from water dropwort and Arabidopsis. The TPS family genes in water dropwort were identified by HMMER 3.0 software using PF03936 and PF01397 as queries. The TPS genes of water dropwort and Arabidopsis are represented by different colored shapes, respectively. The phylogenetic tree was constructed by MEGA 7.0 using neighbor-joining method with 1,000 bootstraps.

TPS is the key enzyme in the terpenoid biosynthesis pathway and contributes to the formation of various terpenoid backbones (Bohlmann and Keeling, 2008). In this study, 25 TPS family genes were identified from the transcriptome of water dropwort. The phylogenetic tree was constructed using the TPS proteins from water dropwort and Arabidopsis. These TPS family genes were divided into five subfamilies, including TPS-a, TPS-b, TPS-c, TPS-e/f, and TPS-g (Figure 4). TPS-b subfamily has 13 TPS members in water dropwort, which accounts the highest proportion among all subfamilies.

### Analysis of Differentially Expressed Genes

**FIGURE 5:**
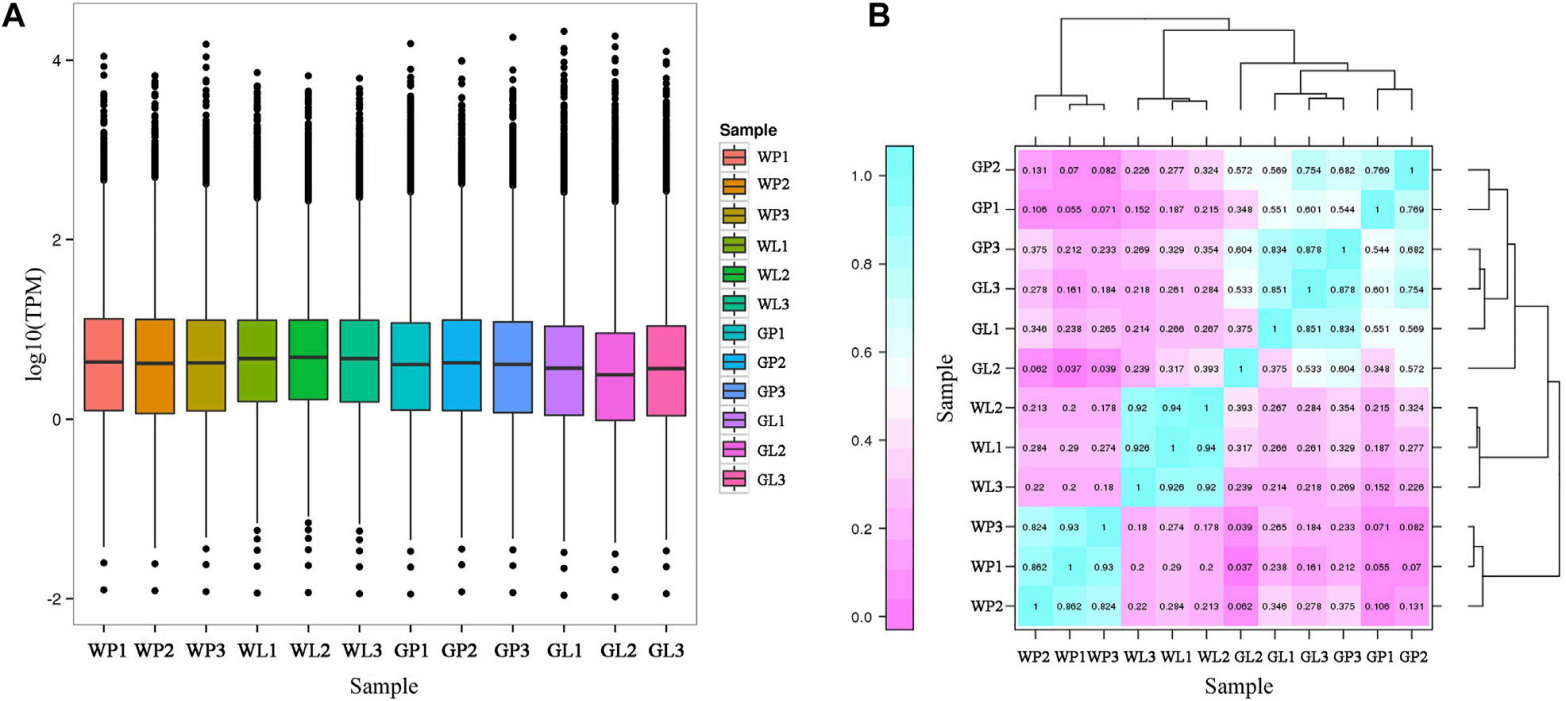
The statistic of TPM values among all water dropwort samples. **(A)** The distribution of TPM values in different water dropwort samples. **(B)**The heat map for correlation analysis of expression levels in different water dropwort samples. GP: green petioles; GL: green leaf blades; WP: white petioles; WL: white leaf blade.

The TPM values were used for visualization and correlation analysis. The distribution of TPM values in all samples were shown in Figure 5A. The correlation analysis of expression levels in different samples was conducted (Figure 5B). As the petioles of water dropwort were the main edible organs, the DEGs analysis was conducted in GP and WP. In this study, 5,288 differentially expressed genes (DEGs) were identified between GP and WP, including 1,253 up-regulated and 4,035 down-regulated DEGs (Supplementary Figure S2).

**FIGURE 6:**
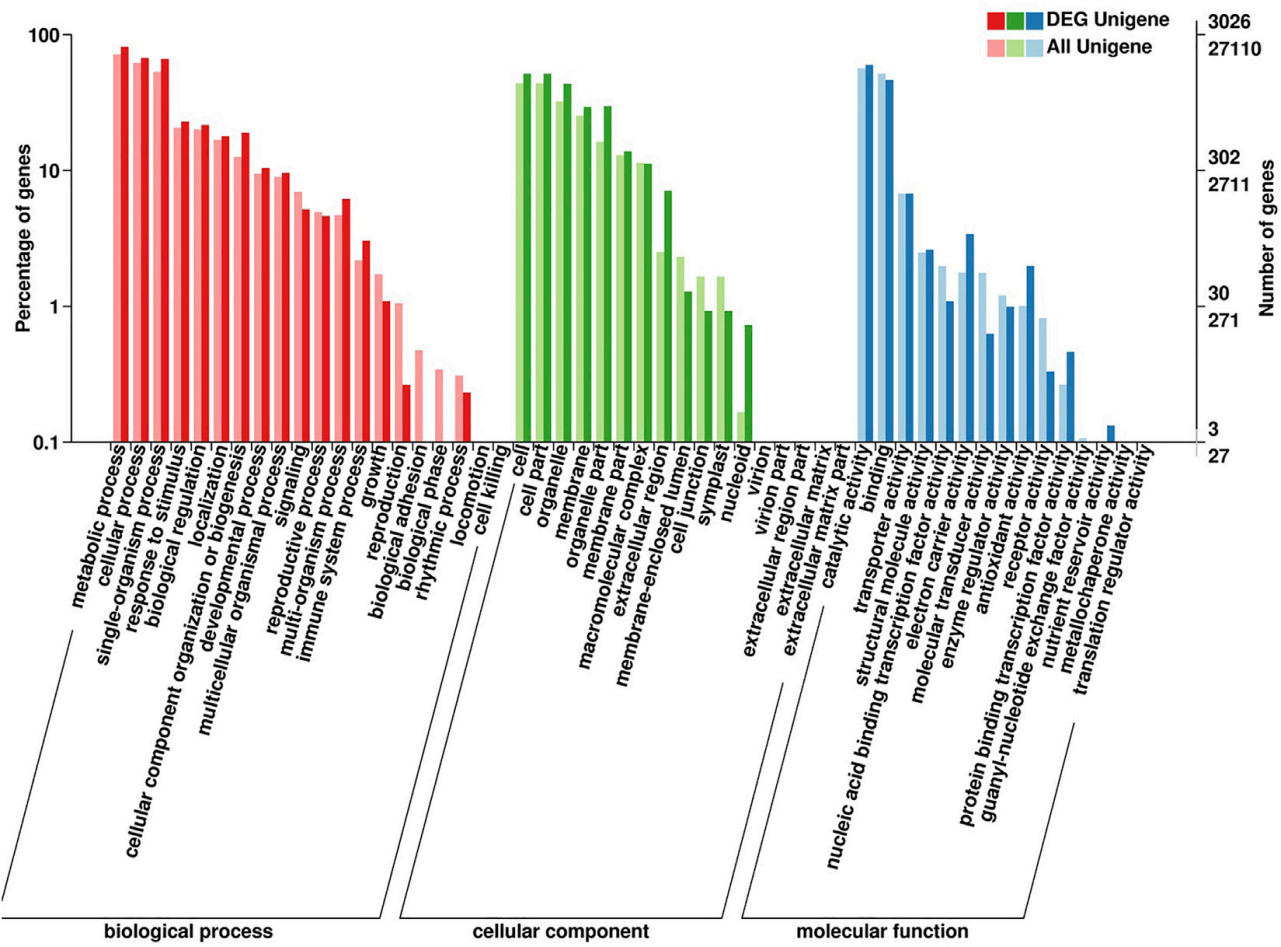
GO enrichment analysis of DEGs. Each gene was classified into at least one GO term, and all these genes were grouped into three categories, namely, molecular function, cellular component, and biological process.

The GO annotation and enrichment analysis suggested that the majority of DEGs were classified into three categories: “biological process,” “cellular component” and “molecular function” (Figure 6). As for ‘biological process’ category, the DEGs were the most enriched in ‘metabolic process’. The results indicated that the DEGs involved in metabolites biosynthesis play important roles in shading treatment of water dropwort. In order to investigate the DEGs of various metabolites pathways in water dropwort, the KEGG analysis was conducted. Carbon metabolism is the metabolic pathway with the most DEGs in photosynthetic organisms, followed by carbon fixation (Supplementary Figure S3). After the shading treatment, the volatile compound contents of water dropwort were decreased, and its flavor improved significantly. A total of 22 DEGs were enriched in terpenoid backbone biosynthesis. We speculated that these DEGs were involved in the regulation of terpenoid biosynthesis in water dropwort.

### Transcription Profiles of Genes Involved in Terpenoids Biosynthesis

**FIGURE 7:**
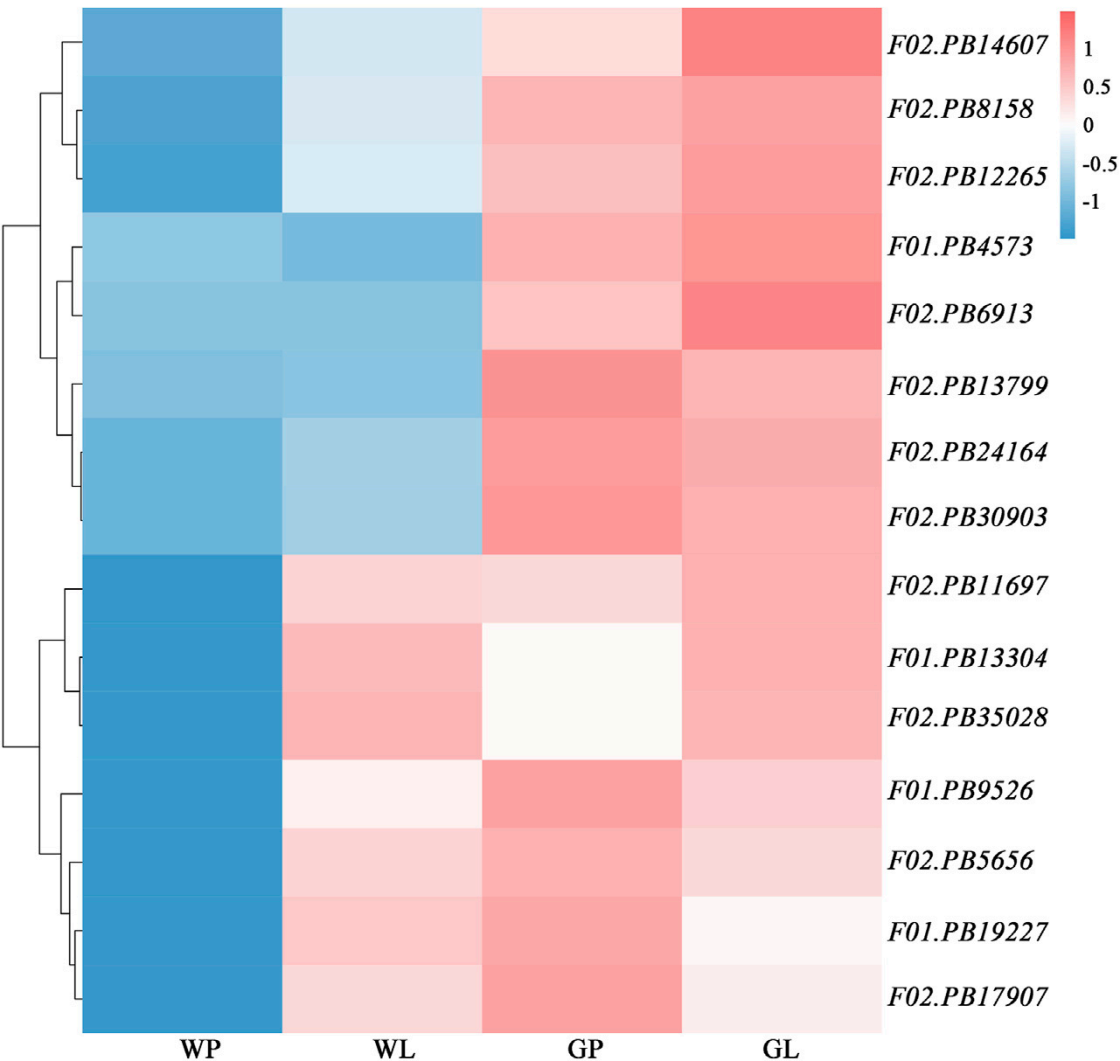
Heatmap of DEGs transcript abundances in terpenoids backbone biosynthesis pathway in water dropwort. GP: green petioles; GL: green leaf blades; WP: white petioles; WL: white leaf blade. Red and blue colors represent high and low transcript abundances, respectively.

**FIGURE 8:**
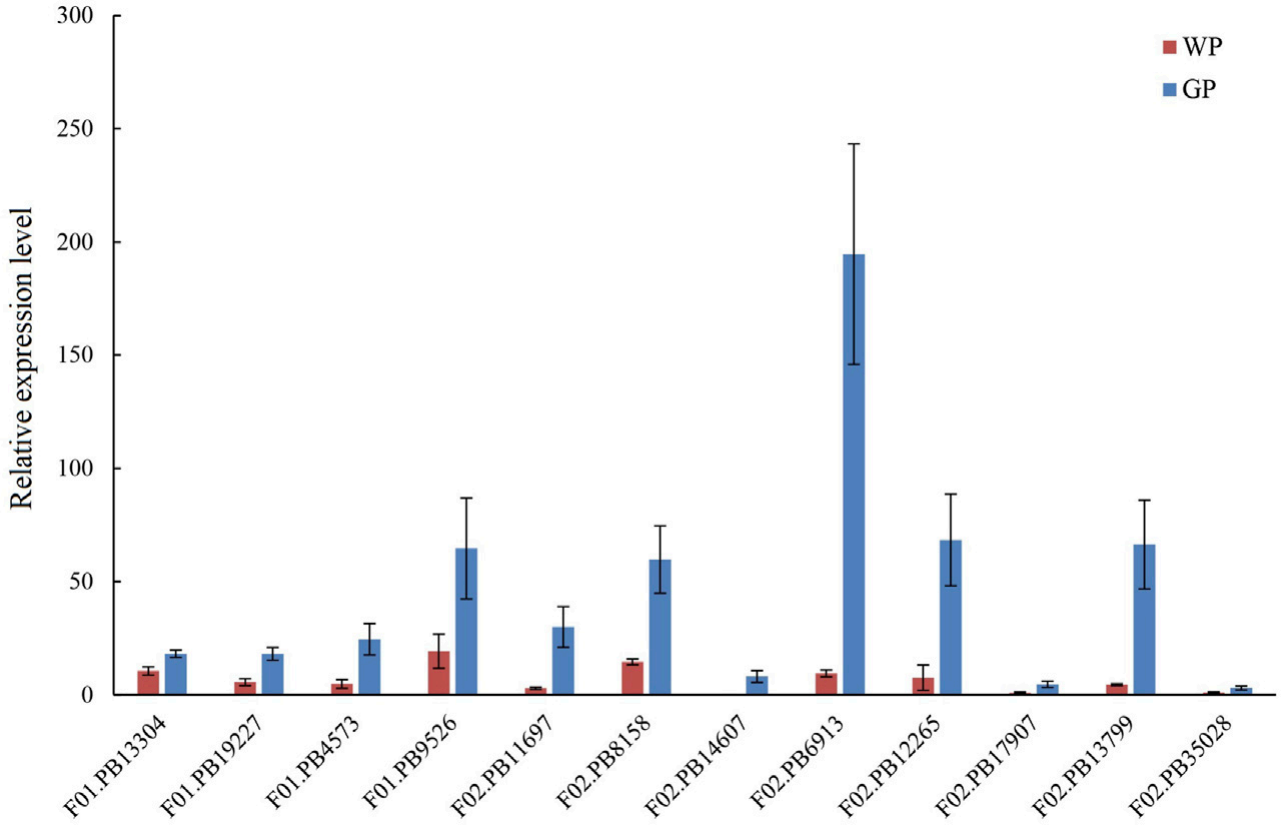
qRT-PCR assay of selected DEGs in terpenoids backbone biosynthesis pathway in water dropwort. The bar represents the mean values of three independent experiments ±SD. WP: white petioles; GP: green petioles.

The volatile terpenoids of water dropwort were decreased after the shading treatment. We analyzed the transcription profiles of DEGs in the terpenoids backbone biosynthesis pathway. The results indicated that the transcription levels of the DEGs were down-regulated after the shading treatment (Figure 7). To further verify the reliability of transcriptome data, 12 DEGs including *DXS* (F01.PB13304), *HDS* (F01.PB19227), *HDR* (F01.PB4573), *GGPS* (F01.PB9526, F02.PB11697, and F02.PB8158), *SPS* (F02.PB14607, F02.PB6913), and *TPS* (F02.PB12265, F02.PB17907, F02.PB13799, and F02.PB35028) in terpenoid biosynthesis pathway were selected to perform qRT-PCR assay (Figure 8). The expression levels of terpenoids biosynthesis genes in white petioles were down-regulated compared to green petioles, which were consistent with the results of RNA-Seq. Pearson’s correlation analysis indicated that the expression levels of terpenoids biosynthesis genes were positively correlated with the volatile contents in water dropwort (Supplementary Table S9).

## Discussion

In view of the rising importance of aquatic vegetables, more and more studies on water dropwort have been reported in recent years (Park et al., 2015; Gao et al., 2020). However, the research on molecular mechanisms in water dropwort is still lagging far behind other crop species. In this work, the full-length transcriptome of water dropwort was constructed by the integrative analysis of the PacBio SMRT and Illumina RNA sequencing. A total of 57,743 non-redundant transcripts were obtained from the full-length transcriptome. The SSR, lncRNAs, and AS events were analyzed based on the full-length transcriptome. SSR is also known as microsatellites, which is a type of short tandem repetitive DNA sequences with 1–6 bases pairs (Toth et al., 2000). SSRs have been widely applied in population genetics, genetic linkage mapping, and molecular breeding (Kumar et al., 2015; Nachimuthu et al., 2015). A total of 28,514 SSRs were identified from the non-redundant transcripts, and the mono-nucleotide repeats were the most abundant SSRs. LncRNAs are a kind of non-coding RNA, which play important roles in biological processes by acting on target genes (Wang and Chang, 2011). Based on CPC, CNCI, CPAT, and Pfam databases, 612 lncRNAs were recognized and their target genes were predicted. The SSRs and lncRNAs obtained from the full-length transcriptome will provide useful information for future research on water dropwort.

Water dropwort was a popular vegetable in East Asia on account of its nutritional values and distinctive flavor (Ji et al., 2021). By using SPME-GC-O technology, terpenoids were investigated to be the characteristic aroma components in water dropwort. Terpenoids are widely existed in plants tissues and contribute to the formation of distinct smells (Dudareva et al., 2004). Water dropwort with a high concentration of terpenoids will emit undesirable flavor (Seo and Baek, 2005). Shading treatment was an effective approach to improve the flavor in the production of water dropwort. Whereas now, the changes of terpenoids and related molecular mechanism during shading treatment in water dropwort remains unknown. Current results demonstrated that the volatile compound contents of water dropwort were significantly decreased after shading treatment. Altered volatile substance content was related to the flavor improvement of water dropwort. Similarly, the high levels of flavonols resulted in the bitter flavor of tea, and the flavonols contents of tea were increased under UV-B and decreased after shading treatments (Zhao et al., 2021).

The key regulatory genes and related molecular mechanisms play an important role in plant growth and terpenoids biosynthesis (Feng et al., 2020; Nagegowda and Gupta, 2020). The DEGs analysis was conducted to investigate the molecular mechanism of water dropwort during shading treatment. In “biological process” category, the DEGs were the most enriched in “metabolic process,” suggesting the regulation of metabolites biosynthesis is of great importance in water dropwort during shading treatment (Young et al., 2010). KEGG analysis showed that DEGs in photosynthesis-related pathways resulted in the albino of water dropwort, which were induced by the lack of light after shading treatment. Previous study also reported that the photosynthetic pathway and chlorophyll content of *Tetrastigma hemsleyanum* Diels et Gilg were affected by shading treatment (Dai et al., 2009). A total of 22 DEGs were enriched in terpenoid backbone biosynthesis pathway based on the transcriptome analysis, suggesting these DEGs were involved in the terpenoids biosynthesis in water dropwort. Integrative analysis of transcriptome and metabolome demonstrated that the biosynthesis of sesquiterpene in *Sindora glabra* was regulated by the DEGs in terpenoid backbone biosynthesis pathway (Yu et al., 2021).

Terpenoids were synthesized using C_5_ isoprene as substrate under the catalysis of various enzymes in plants (Nagegowda, 2010). At present, the genes related to terpenoids biosynthesis in water dropwort are still unclear. In this work, qRT-PCR assay verified that the DEGs identified from transcriptome were down-regulated after shading treatment, such as *DXS* (F01.PB13304), *HDS* (F01.PB19227), *HDR* (F01.PB4573), and *TPS* (F02.PB12265, F02.PB17907, F02.PB13799, and F02.PB35028). DXS is the first rate-limiting enzyme in MEP pathway, which catalyze the condensation of glyceraldehyde 3-phosphate (G3P) and pyruvate to form 1-deoxy-d-xylulose-5-phosphate (DXP) (Rohdich et al., 2003). The DXS has been reported in pepper, rubber tree, potato, and masson pine (Bouvier et al., 1998; Seetang-Nun et al., 2008; Henriquez et al., 2016; Li et al., 2021). HDS is 1-hydroxy-2-methyl-2-(*E*)-butenyl-4-diphosphate synthase, which is responsible for the biosynthesis of 1-hydroxy-2-methyl-2-(*E*)-butenyl-4-diphosphate (HMBPP) (Gutierrez-Nava et al., 2004). In the last step of MEP pathway, HDR catalyzed the conversion of HMBPP into IPP and DMAPP (Laule et al., 2003). The polyprenyl diphosphates generated from IPP and DMAPP was further converted into various terpenoid backbones by the enzymes of known TPS family (Bohlmann et al., 1998; Huang et al., 2017). In the future works, the above DEGs associated terpenoids biosynthesis will be selected for further functional verification in water dropwort.

## Conclusion

In conclusion, we constructed the full-length transcriptome and obtained 57,743 non-redundant transcripts from water dropwort. To the best of our knowledge, this study is the first to report a full-length transcriptome in water dropwort. The AS events, SSR, and lncRNAs were predicted based on the transcriptome. The volatile compound contents of water dropwort were decreased after the shading treatment. 22 DEGs in terpenoid backbone biosynthesis pathway were differentially expressed. The current study identified the terpenoids biosynthesis genes and will be helpful for investigating the mechanism of terpenoids biosynthesis in water dropwort. Also, the full-length transcriptome in our study will establish a basis for functional genomics and genetic engineering breeding in water dropwort in the future.

## Data Availability

The original contributions presented in the study are publicly available. This data can be found here: PRJNA780322.' 211112 SL: Assessed for PM, proceeding with review
