# Supplementary material for: Integrative Analysis of Long- and Short-Read Transcriptomes Identify the Regulation of Terpenoids Biosynthesis Under Shading Cultivation in Oenanthe javanica
Source: Front Genet. 2022 Apr 7;13:813216. doi: 10.3389/fgene.2022.813216 (PMC9022222; doi:10.3389/fgene.2022.813216)
Supplement: Supplementary file 3 [file DataSheet3.docx]

**Table 8 Statistic of annotated transcripts**

| Annotated database | All | GO | KEGG | KOG | Pfam | Swissprot | COG | eggNOG | NR |
| --- | --- | --- | --- | --- | --- | --- | --- | --- | --- |
| Annotated number | 57,118 | 27,110 | 25,644 | 37,227 | 48,433 | 43,401 | 25,353 | 55,815 | 56,684 |

**Table 9 Pearson’s correlation analysis between gene expression and volatile contents in the petioles water dropwort**

| Genes | correlation coefficient |
| --- | --- |
| *F01.PB13304* | 0.988** |
| *F01.PB19227* | 0.932** |
| *F01.PB4573* | 0.913* |
| *F02.PB5656* | 0.991** |
| *F01.PB9526* | 0.981** |
| *F02.PB11697* | 0.949** |
| *F02.PB8158* | 0.973** |
| *F02.PB14607* | 0.914* |
| *F02.PB6913* | 0.823* |
| *F02.PB12265* | 0.926** |
| *F02.PB17907* | 0.922** |
| *F02.PB13799* | 0.97** |
| *F02.PB24164* | 0.87* |
| *F02.PB30903* | 0.862* |
| *F02.PB35028* | 0.947** |

* and ** indicates significance at *P* < 0.05 and *P* < 0.01, respectively.
