# Supplementary material for: Integrative Analysis of Long- and Short-Read Transcriptomes Identify the Regulation of Terpenoids Biosynthesis Under Shading Cultivation in Oenanthe javanica
Source: Front Genet. 2022 Apr 7;13:813216. doi: 10.3389/fgene.2022.813216 (PMC9022222; doi:10.3389/fgene.2022.813216)
Supplement: Supplementary file 5 [file DataSheet2.docx]

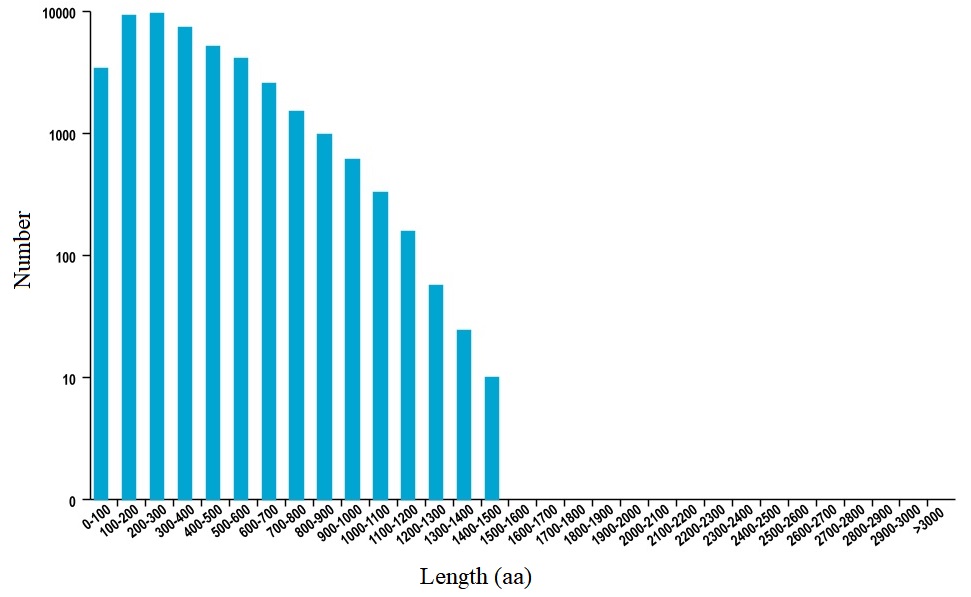


**Supplementary Figure 1** The length distribution of protein encoded by CDS.


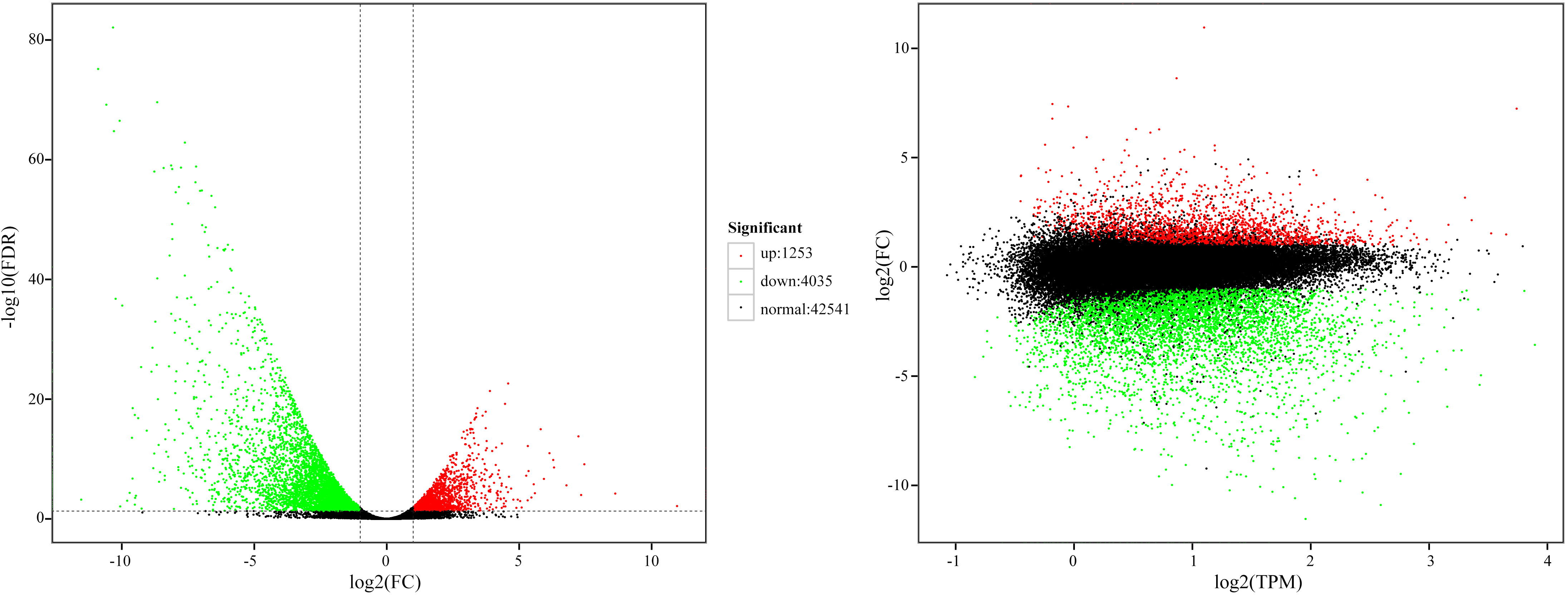


**Supplementary Figure 2** The volcano plot and MA plot of DEGs between green petiole and white petiole.


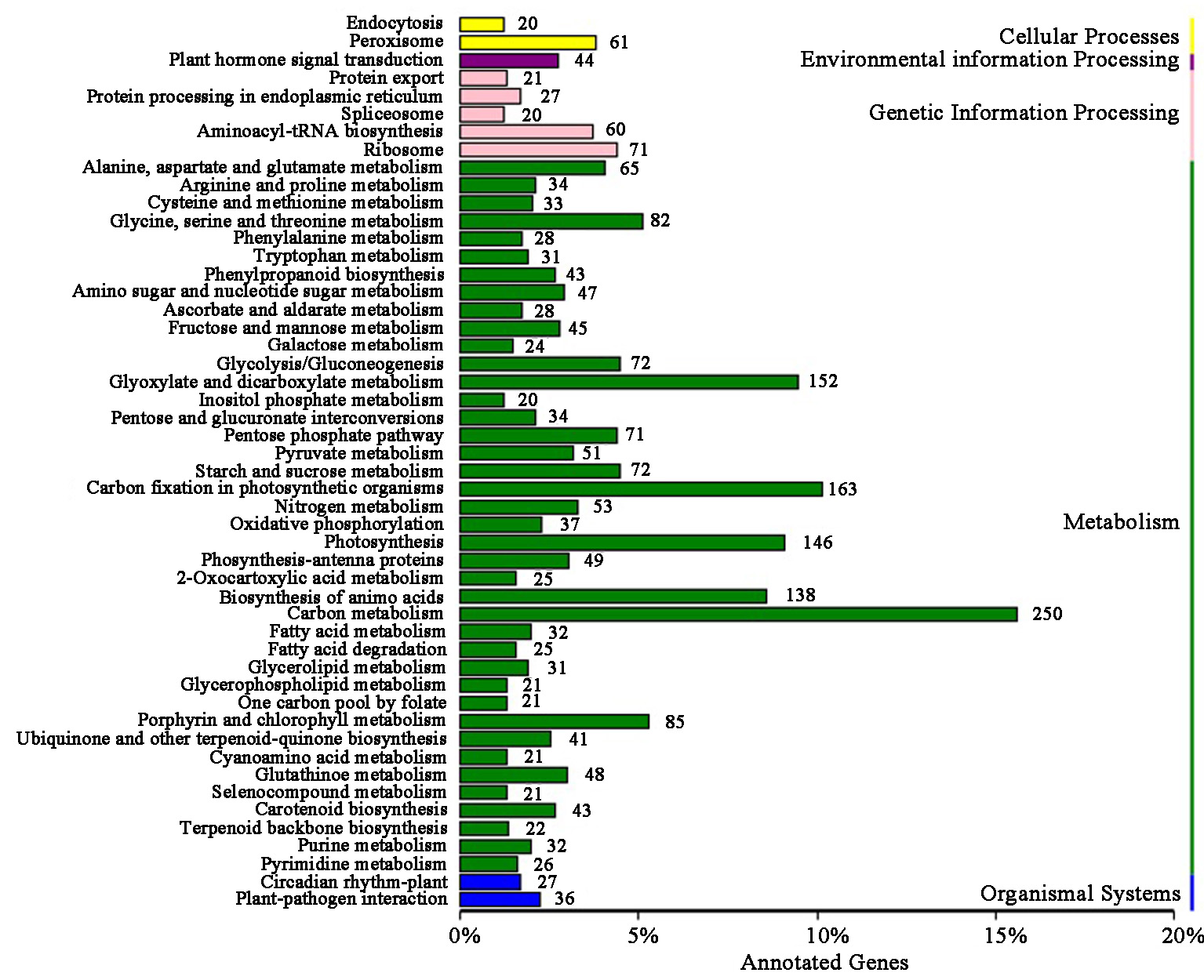


**Supplementary Figure 3** Differentially expressed genes enriched on different KEGG pathways.
