# Supplementary material for: Integrative Analysis of Long- and Short-Read Transcriptomes Identify the Regulation of Terpenoids Biosynthesis Under Shading Cultivation in Oenanthe javanica
Source: Front Genet. 2022 Apr 7;13:813216. doi: 10.3389/fgene.2022.813216 (PMC9022222; doi:10.3389/fgene.2022.813216)
Supplement: Supplementary file 6 [file DataSheet1.docx]

**Table 1 The primer sequences used in qRT-PCR**

| Gene ID | Forward primer (5’-3’) | Reverse primer (5’-3’) |
| --- | --- | --- |
| F01.PB13304 | GTTGGTCATCAGTCATATCCGCACAA | GTCATAATCACTCTCCGCACGCTTAG |
| F01.PB19227 | GGTGATGGTAGGCAATGTGGCTATC | ACAGTTATGCGAACAATATCTGCTCCT |
| F01.PB4573 | GGAACTCAAGCAACACATCACATCTAC | ACCAATTCTCTTGTCACTGTCAATCCA |
| F01.PB9526 | GCTGTCGGAGTGTTATATCAGGTTGTT | CTCTGCCACTTCCATTGCCTTCTC |
| F02.PB11697 | TGCTCTGTATCGCCGCCTGT | CGTCCTCGCCGTAGACCTTATG |
| F02.PB8158 | CTGATATGGCGATTCTTGCTGGTGAT | GCTATGACTCTTAGAAGACGGTCCTCT |
| F02.PB14607 | AGACTGGTTCACTTGATGAGGCTATTG | AAGGAACTAGGAGGAAGGCATTGGA |
| F02.PB6913 | AGACTGGTTCGTTGGATGAGGCTAT | ATCGGAAGGCACTCTGAGGAAGG |
| F02.PB12265 | TAGCCGCCTCGTTGAAGTTGAAG | GGTAGCCAAGTCCTAATCGTTCTATGT |
| F02.PB17907 | GGTGCGTCGTCGATCCAATGTAT | TTGCCGAGTAGTTGAGGTATGGTTG |
| F02.PB13799 | ACATAGAACGATTAGGACTTGGCTACC | GCAGTAGTGTGAAGGCTCTTGTGA |
| F02.PB35028 | GCTCATCTCTGCATGGTTGACACT | TGTTCATCGTCCTGTATGTTTCATCCA |

**Table 2 The statistics of Illumina sequencing**

| Sample | ReadSum | BaseSum | GC(%) | Q30(%) |
| --- | --- | --- | --- | --- |
| WP1 | 21,956,912 | 6,544,485,628 | 42.83 | 92.37 |
| WP2 | 23,020,103 | 6,872,892,728 | 42.65 | 92.36 |
| WP3 | 20,788,074 | 6,209,808,512 | 42.99 | 92.22 |
| WL1 | 21,494,147 | 6,417,054,642 | 42.74 | 91.63 |
| WL2 | 23,776,541 | 7,103,270,102 | 42.68 | 92.29 |
| WL3 | 26,143,054 | 7,804,031,942 | 42.46 | 91.7 |
| GP1 | 25,054,007 | 7,485,029,496 | 42.52 | 92.35 |
| GP2 | 20,408,790 | 6,086,065,280 | 42.86 | 92.44 |
| GP3 | 30,750,204 | 9,189,531,702 | 42.76 | 92.76 |
| GL1 | 21,326,859 | 6,369,264,436 | 42.91 | 92.16 |
| GL2 | 21,785,856 | 6,508,467,740 | 43.03 | 92.14 |
| GL3 | 21,064,052 | 6,287,840,672 | 42.9 | 92.54 |

**Table 3 Statistics of reads of insert (ROI)**

| Samples | cDNA size | Reads of insert | Read bases of insert | Mean read length of insert | Mean read quality of insert | Mean number of passes |
| --- | --- | --- | --- | --- | --- | --- |
| F01 | 1-2K | 70,011 | 173,454,783 | 2,477 | 0.93 | 14.00 |
| F01 | 2-3K | 66,082 | 264,006,820 | 3,995 | 0.91 | 8.00 |
| F01 | 3-6K | 58,024 | 242,561,026 | 4,180 | 0.90 | 7.00 |
| F01 | All | 194,117 | 680,022,629 | 3,502 | 0.91 | 9.00 |
| F02 | 1-2K | 69,924 | 208,570,790 | 2,982 | 0.91 | 11.00 |
| F02 | 2-3K | 108,769 | 338,381,662 | 3,111 | 0.91 | 8.00 |
| F02 | 3-6K | 76,108 | 255,680,456 | 3,359 | 0.91 | 10.00 |
| F02 | All | 254,801 | 802,632,908 | 3,149 | 0.91 | 9.00 |

**Table 4 The statistics of consensus isoforms by ICE software**

| Samples | Size | Number of consensus isoforms | Average consensus isoforms read length | Number of polished high-quality isoforms | Number of polished low-quality isoforms | Percent of polished high-quality isoforms (%) |
| --- | --- | --- | --- | --- | --- | --- |
| F01 | 0-1 kb | 3,492 | 901 | 3,265 | 227 | 93.50% |
|  | 1-2 kb | 16,688 | 1,403 | 14,859 | 1,829 | 89.04% |
|  | 2-3 kb | 12,988 | 2,353 | 10,854 | 2,134 | 83.57% |
|  | 3-6 kb | 13,558 | 3,632 | 9,190 | 4,368 | 67.78% |
|  | > 6 kb | 833 | 9,944 | 18 | 815 | 2.16% |
|  | All | 47,559 | 2,410 | 38,186 | 9,373 | 80.29% |
| F02 | 0-1 kb | 3,409 | 821 | 3,199 | 209 | 93.84% |
|  | 1-2 kb | 19,258 | 1,502 | 17,238 | 2,020 | 89.51% |
|  | 2-3 kb | 25,166 | 2,394 | 20,162 | 5,004 | 80.12% |
|  | 3-6 kb | 11,104 | 3,474 | 7,487 | 3,617 | 67.43% |
|  | > 6 kb | 883 | 9,521 | 10 | 873 | 1.13% |
|  | All | 59,820 | 2,322 | 48,096 | 11,723 | 80.40% |
